# JAK2-IGF1 axis in osteoclasts regulates postnatal growth in mice

**DOI:** 10.1172/jci.insight.137045

**Published:** 2021-03-08

**Authors:** David W. Dodington, Jenalyn L. Yumol, Jiaqi Yang, Evan Pollock-Tahiri, Tharini Sivasubramaniyam, Sandra M. Sacco, Stephanie A. Schroer, Yujin E. Li, Helen Le, Wendy E. Ward, Minna Woo

**Affiliations:** 1 Toronto General Hospital Research Institute, University Health Network, Toronto, Ontario, Canada.; 2 Department of Kinesiology and; 3 Department of Health Sciences, Brock University, St. Catharines, Ontario, Canada.; 4 Institute of Medical Science and Department of Immunology and; 5 Division of Endocrinology and Metabolism, Department of Medicine, University Health Network/Sinai Health System, University of Toronto, Toronto, Ontario, Canada.

**Keywords:** Bone Biology, Endocrinology, Bone development, Growth factors, Osteoclast/osteoblast biology

## Abstract

Osteoclasts are specialized cells of the hematopoietic lineage that are responsible for bone resorption and play a critical role in musculoskeletal disease. JAK2 is a key mediator of cytokine and growth factor signaling; however, its role in osteoclasts in vivo has yet to be investigated. To elucidate the role of JAK2 in osteoclasts, we generated an osteoclast-specific JAK2–KO (Oc-JAK2–KO) mouse using the Cre/Lox-P system. Oc-JAK2–KO mice demonstrated marked postnatal growth restriction; however, this was not associated with significant changes in bone density, microarchitecture, or strength, indicating that the observed phenotype was not due to alterations in canonical osteoclast function. Interestingly, Oc-JAK2–KO mice had reduced osteoclast-specific expression of IGF1, suggesting a role for osteoclast-derived IGF1 in determination of body size. To directly assess the role of osteoclast-derived IGF1, we generated an osteoclast-specific IGF1–KO mouse, which showed a similar growth-restricted phenotype. Lastly, overexpression of circulating IGF1 by human transgene rescued the growth defects in Oc-JAK2–KO mice, in keeping with a causal role of IGF1 in these models. Together, our data show a potentially novel role for Oc-JAK2 and IGF1 in the determination of body size, which is independent of osteoclast resorptive function.

## Introduction

Osteoclasts are specialized cells of the hematopoietic lineage and mostly known for their function in bone resorption (1). Osteoclasts play a critical role in the pathogenesis of several diseases, including osteoporosis (2), rheumatoid arthritis (3), periodontal disease (4), myeloma (5), and metastatic cancer (6); thus, there is an increasing need to elucidate the mechanisms of osteoclast function under homeostatic conditions in order to better understand the pathogenesis of osteoclast-related diseases and develop novel therapeutic strategies.

Janus kinase 2 (JAK2) is a ubiquitously expressed tyrosine kinase involved in multiple signal transduction pathways (7). JAK2 is responsible for transmitting extracellular signals from various cytokines and growth factors, including growth hormone, erythropoietin, thrombopoietin, prolactin, IL-6, granulocyte-macrophage CSF (GM-CSF), and IFN-γ. JAK2 is also a clinically relevant gene due to the causal link between activating JAK2 mutations and myeloproliferative disorders (8). In bone, there is some evidence to suggest a role for JAK2 in osteoclast biology. Osteoclasts derived from individuals with myelofibrosis harboring JAK2 activating mutations have impaired osteolytic capacity, implicating JAK2 with osteoclast dysfunction (9). In vitro studies using JAK2 inhibitors have also highlighted a potential role for JAK2 in osteoclasts. Osteoblast/osteoclast cocultures treated with the JAK1/2 inhibitor baricitinib showed decreased osteoclastogenesis due to reduced RANKL expression in osteoblasts (10). In another study, use of the JAK2 inhibitor AG490 in purified osteoclast cultures inhibited osteoclast apoptosis in response to RANKL withdrawal (11). Despite these data, the role of osteoclast JAK2 in vivo has yet to be investigated.

The objective of this study was to elucidate the in vivo function of JAK2 in osteoclasts. To accomplish this, we used the Cre/Lox-P system to generate an osteoclast-specific JAK2–KO (Oc-JAK2–KO) mouse. In this study, we found that Oc-JAK2–KO mice had marked postnatal growth restriction; however, this was not associated with significant changes in bone mineral density (BMD), bone microarchitecture, bone strength, osteoclast number, or osteoclastic gene expression, suggesting that osteoclast JAK2 modulates body size without altering the balance between bone formation and bone resorption. Interestingly, loss of JAK2 impaired expression of IGF1 in osteoclasts, suggesting an important role for osteoclast-derived IGF1 in determination of body size. We also show that overexpression of circulating IGF1 in Oc-JAK2–KO mice rescued their growth defects, supporting a causal role between JAK2-mediated IGF1 expression in osteoclasts and postnatal growth.

## Results

### Generation of Oc-JAK2–KO mice.

**Figure 1:**
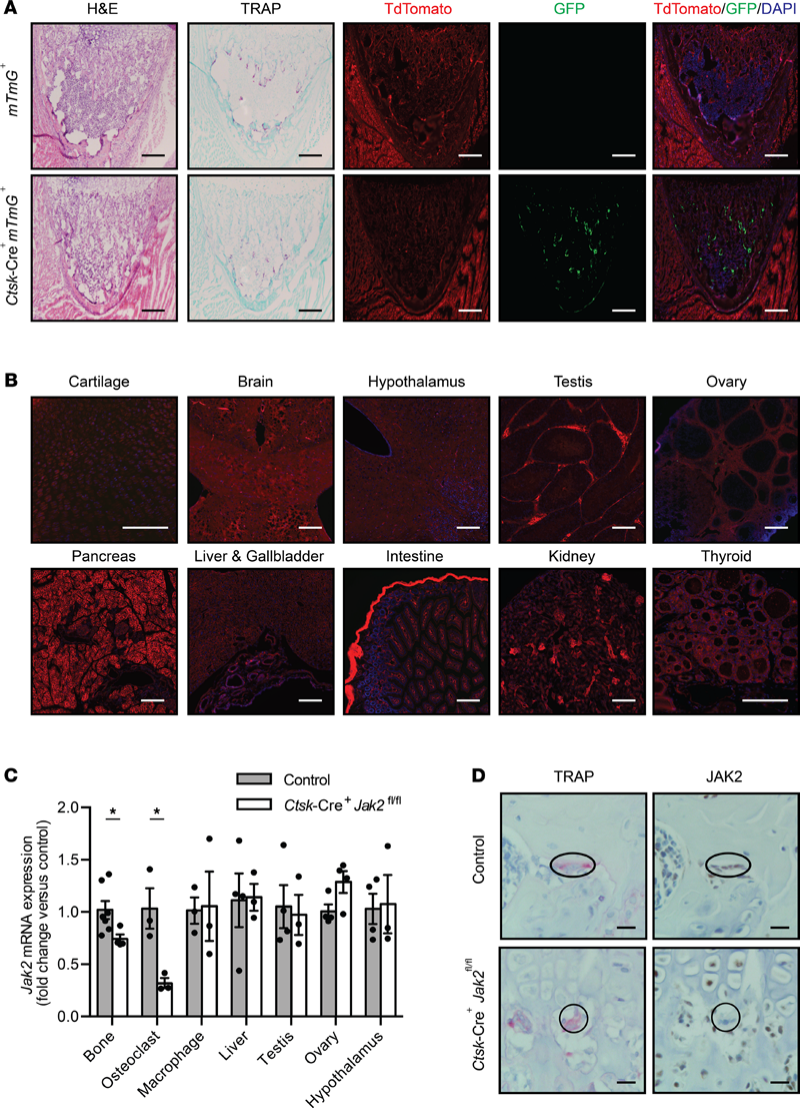
Generation of osteoclast-specific JAK2–KO mice. *Ctsk*-Cre–expressing mice were bred to *mTmG* mice or mice with floxed *Jak2* gene to generate reporter and osteoclast-specific JAK2–KO mice. (**A**) Representative images of knee joint from *mTmG*^+^ (*n* = 3) and *Ctsk*-Cre^+^*mTmG*^+^ mice (*n* = 3) at 1 week of age stained for H&E, tartrate resistant alkaline phosphatase (TRAP), and imaged for fluorescence of endogenous Cre reporter activity. Staining/imaging was performed on adjacent serial frozen sections. Scale bars: 100 μm. (**B**) Representative fluorescence images of cartilage (femur growth plate), brain, hypothalamus, testis, ovary, pancreas, liver/gallbladder, small intestine, kidney, and thyroid from *Ctsk*-Cre^+^*mTmG*^+^ mice (*n* = 3). Tissues were collected from 8-week-old mice, except for growth plates, which were collected from 1-week-old mice. Scale bars: 100 μm. (**C**) *Jak2* mRNA expression in bone [*n* = 7 (control), *n* = 4 (*Ctsk*-Cre^+^*Jak2^fl/fl^*)], BM-derived osteoclasts [*n* = 3 (control), *n* = 3 (*Ctsk*-Cre^+^*Jak2^fl/fl^*)], BM-derived macrophages [*n* = 3 (control), *n* = 3 (*Ctsk*-Cre^+^*Jak2^fl/fl^*)], liver [*n* = 4 (control), *n* = 3 (*Ctsk*-Cre^+^*Jak*2*^fl/fl^*)], testis [*n* = 4 (control), *n* = 3 (*Ctsk*-Cre^+^*Jak2^fl/fl^*)], ovary [*n* = 4 (control), *n* = 4 (*Ctsk*-Cre^+^*Jak2^fl/fl^*)], and hypothalamus [*n* = 4 (control), *n* = 3 (*Ctsk*-Cre^+^*Jak2^fl/fl^*)] from control and *Ctsk*-Cre^+^*Jak2^fl/fl^* mice. Values are normalized to *18S* mRNA levels and presented as fold change over control group. (**D**) Representative images of femur from control (*n* = 3) and *Ctsk*-Cre^+^*Jak2^fl/fl^* mice (*n* = 3) stained for TRAP and immunostained for JAK2. Staining was performed on adjacent serial sections. The circle highlights TRAP^+^ multinucleated giant cells. Scale bars: 10 μm. Data represent mean ± SEM. Differences between groups were analyzed for statistical significance by Student’s unpaired *t* test; *P < 0.05. Oval highlights TRAP^+^ multinucleated giant cells.

To validate the osteoclast specificity of the *Cathepsin K*-Cre (*Ctsk*-Cre) mouse, we bred *Ctsk*-Cre^+^ mice to *mTmG^+/–^* reporter mice whose tissue expressed either fluorescence TdTomato in the absence of Cre-mediated recombination or enhanced GFP (EGFP) in its presence. Sections of the knee joint showed robust expression of EGFP along the surface of the trabecular bone in *Ctsk*-Cre^+^*mTmG^+^* mice (Figure 1A). Staining for tartrate resistant alkaline phosphatase (TRAP) on the adjacent serial section confirmed that EGFP was present in the expected distribution for osteoclasts. EGFP was not detected in Cre^–^ controls. Sections of other tissues, including growth plate cartilage, brain, hypothalamus, testis, ovary, pancreas, liver, small intestine, kidney, and thyroid did not express EGFP (Figure 1B). Next, to generate Oc-JAK2–KO mice, *Ctsk*-Cre^+^ mice were bred to *Jak2* floxed mice (*Jak2^fl/fl^*) and resultant *Ctsk*-Cre^+^*Jak2^fl/+^* mice were crossed to *Jak2^fl/fl^* mice to generate KO mice (*Ctsk*-Cre^+^*Jak2^fl/fl^*) and littermate control mice (*Jak2^fl/fl^* and *Ctsk*-Cre^+^*Jak2^fl/+^*). To validate osteoclast-specific deficiency of JAK2 in our Oc-JAK2–KO mouse, we measured *Jak2* mRNA levels in tissues and in BM-derived osteoclasts from *Ctsk*-Cre^+^*Jak2^fl/fl^* and *Jak^fl/fl^* mice. JAK2 mRNA expression was reduced by 26% in bone and by 70% in osteoclast cultures from *Ctsk*-Cre^+^*Jak2^fl/fl^* mice (Figure 1C). Importantly, *Jak2* mRNA expression was unchanged in BM-derived macrophages, liver, testis, ovary, or hypothalamus. There have been previous reports of *Ctsk*-Cre mediating germline gene deletion of certain floxed alleles (12); however, this did not occur in *Ctsk*-Cre^+^*Jak2^fl/fl^* mice, since *Jak2* expression was preserved in nonosteoclast cells and tissues. Lastly, we performed IHC for JAK2 on femur sections with TRAP staining on the adjacent serial section to identify TRAP^+^ multinucleated giant cells. In control mice, there was weak to moderate immunostaining for JAK2 in TRAP^+^ multinucleated giant cells (Figure 1D). In *Ctsk*-Cre^+^*Jak2^fl/fl^* mice, immunoreactivity for JAK2 was absent in TRAP^+^ multinucleated giant cells, with retained expression in other BM cells. Overall, these results indicate that *Ctsk*-Cre^+^*Jak2^fl/fl^* mice represent a rigorous experimental model to study the role of osteoclast JAK2 in vivo. For all experiments, both *Jak2^fl/fl^* and *Ctsk*-Cre^+^*Jak2^fl/+^* mice were used as controls in approximately equal proportions unless otherwise specified. There were no significant differences in any measured outcomes between *Jak2^fl/fl^* and *Ctsk*-Cre^+^*Jak2^fl/+^* mice, indicating that phenotypic differences observed were not to due Cre expression, nor were there any gene dosage effects present with haploinsufficient *Jak2*.

### Ctsk-Cre^+^Jak2^fl/fl^ mice demonstrate postnatal growth restriction.

**Figure 2:**
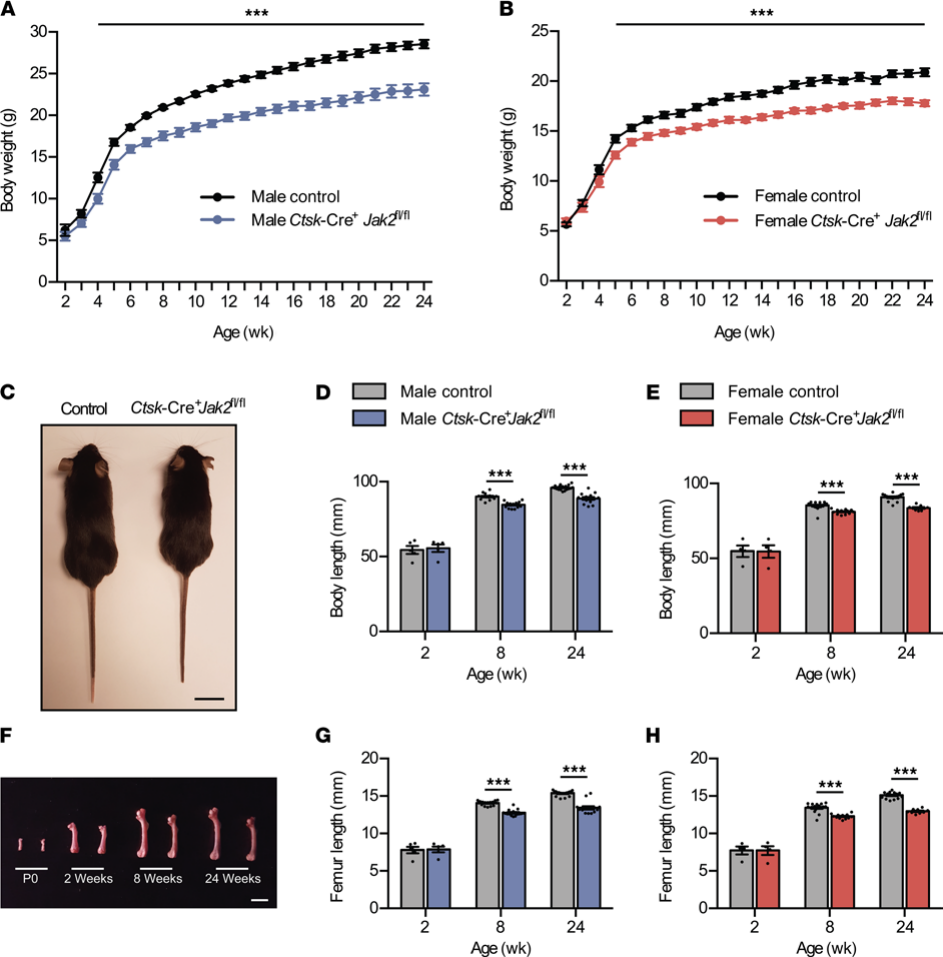
*Ctsk*-Cre^+^*Jak2*^fl/fl^ mice demonstrate postnatal growth restriction. *Ctsk*-Cre^+^*Jak2^fl/fl^* and littermate control mice were monitored until 6 months of age and growth parameters assessed. (**A**) Weekly body weights of male control (*n* = 18) and *Ctsk*-Cre^+^*Jak2^fl/fl^* (*n* = 14) mice. (**B**) Weekly body weights of female control (*n* = 15) and *Ctsk*-Cre^+^*Jak2^fl/fl^* (*n* = 9) mice. (**C**) Representative photograph of 8-week-old male control (left, *n* = 16) and *Ctsk*-Cre^+^*Jak2^fl/fl^* (right, *n* = 15) mice. Scale bar: 20 mm. (**D**) Total body length of male control [*n* = 5 (2 weeks), *n* = 16 (8 weeks), *n* = 18 (24 weeks)] and *Ctsk*-Cre^+^*Jak2^fl/fl^* [*n* = 5 (2 weeks), *n* = 15 (8 weeks), *n* = 14 (24 weeks)] mice. (**E**) Total body length of female control [*n* = 4 (2 weeks), *n* = 18 (8 weeks), *n* = 15 (24 weeks)] and *Ctsk*-Cre^+^*Jak2^fl/fl^* [*n* = 4 (2 weeks), *n* = 12 (8 weeks), *n* = 9 (24 weeks)] mice. (**F**) Representative photograph of femurs from control (left) [*n* = 3 (P0), *n* = 9 (2 weeks), *n* = 34 (8 weeks), *n* = 33 (24 weeks)] and *Ctsk*-Cre^+^*Jak2^fl/fl^* (right) [*n* = 3 (P0), *n* = 9 (2 weeks), *n* = 27 (8 weeks), *n* = 23 (24 weeks)] mice at 0, 2, 8 and 24 weeks of age. Scale bar: 5 mm. (**G**) Femur length of male control [*n* = 5 (2 weeks), *n* = 16 (8 weeks), *n* = 18 (24 weeks)] and *Ctsk*-Cre^+^*Jak2^fl/fl^* [*n* = 5 (2 weeks), *n* = 15 (8 weeks), *n* = 14 (24 weeks)] mice. (**H**) Femur length of female control [*n* = 4 (2 weeks), *n* = 18 (8 weeks), *n* = 15 (24 weeks)] and *Ctsk*-Cre^+^*Jak2^fl/fl^* [*n* = 4 (2 weeks), *n* = 12 (8 weeks), *n* = 9 (24 weeks)] mice. Data represent mean ± SEM. Differences between groups were analyzed for statistical significance by repeated measures ANOVA or 2-way ANOVA; ***P < 0.001.

Control and *Ctsk*-Cre^+^*Jak2^fl/fl^* mice were born in the expected Mendelian ratio and appeared normal at birth, with similar body weights up to 1 month of age. By 4 weeks of age in males (Figure 2A), and 5 weeks of age in females (Figure 2B), *Ctsk*-Cre^+^*Jak2^fl/fl^* mice began to exhibit significantly lower body weight compared with littermate controls. There were no grossly identifiable abnormalities, aside from reductions in overall body size in *Ctsk*-Cre^+^*Jak2^fl/fl^* mice (Figure 2C). Measurement of total body length showed that body length was similar at 2 weeks of age, but a clear distinction in body length between control and *Ctsk*-Cre^+^*Jak2^fl/fl^* mice was observed at 8 and 24 weeks of age (Figure 2, D and E). Similarly, there was an approximate 10% reduction in femur length in both male and female *Ctsk*-Cre^+^*Jak2^fl/fl^* mice compared with littermate controls at 8 and 24 weeks, but not at 2 weeks of age (Figure 2, F–H). Body composition analysis is shown in Table 1. Weights of bone, skeletal muscle, and internal organs were frequently reduced in *Ctsk*-Cre^+^*Jak2^fl/fl^* mice but did not show any significant differences when normalized to body length. Overall, *Ctsk*-Cre^+^*Jak2^fl/fl^* mice demonstrate proportional reduction in body size in the postnatal period.

### Deletion of JAK2 in osteoclasts does not alter bone homeostasis.

**Figure 3:**
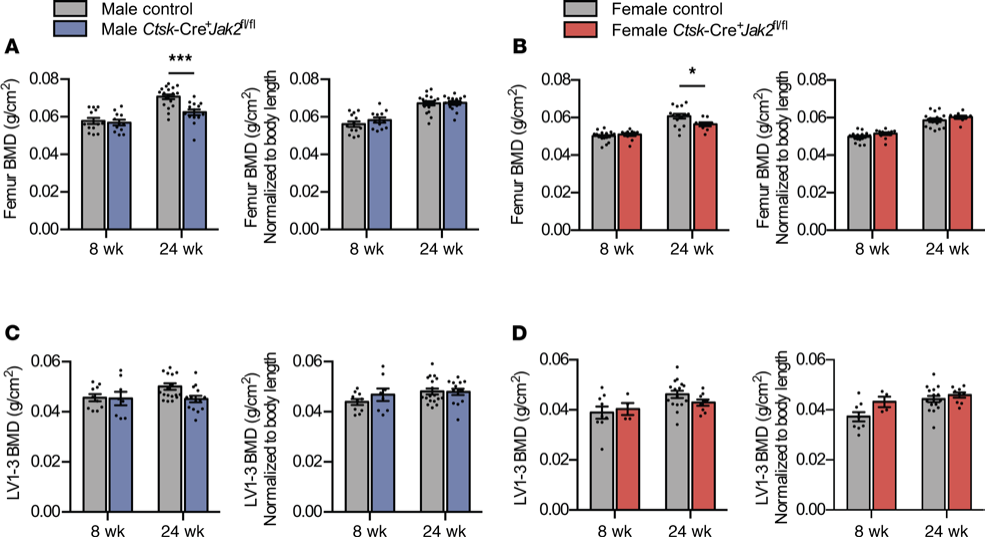
*Ctsk*-Cre^+^*Jak2^fl/fl^* mice have proportional reduction in bone mineral density. Excised femurs and lumbar vertebrae 1-3 (LV1-3) from 8- and 24-week-old *Ctsk*-Cre^+^*Jak2^fl/fl^* and control mice were analyzed by dual x ray absorptiometry (DXA). (**A**) Femur bone mineral density (BMD) (left panel) and BMD normalized to body length (right panel) in male control [*n* = 13 (8 weeks), *n* = 18 (24 weeks)] and *Ctsk*-Cre^+^*Jak2^fl/fl^* [*n* = 12 (8 weeks), *n* = 14 (24 weeks)] mice. (**B**) Femur BMD (left panel) and BMD normalized to body length (right panel) in female control [*n* = 18 (8 weeks), *n* = 16 (24 weeks)] and *Ctsk*-Cre^+^*Jak2^fl/fl^* [*n* = 12 (8 weeks), *n* = 9 (24 weeks)] mice. (**C**) Lumbar vertebrae 1–3 (LV1–3) BMD (left panel) and BMD normalized to body length (right panel) in male control [*n* = 10 (8 weeks), *n* = 18 (24 weeks)] and *Ctsk*-Cre^+^*Jak2^fl/fl^* [*n* = 8 (8 weeks), *n* = 14 (24 weeks)] mice. (**D**) LV1-3 BMD (left panel) and BMD normalized to body length (right panel) in female control [*n* = 9 (8 weeks), *n* = 16 (24 weeks)] and *Ctsk*-Cre^+^*Jak2^fl/fl^* [*n* = 4 (8 weeks), *n* = 9 (24 weeks)] mice. Data represent mean ± SEM. Differences between groups were analyzed for statistical significance by 2-way ANOVA; *P < 0.05, ***P < 0.001.

Next, we sought to determine if targeted deletion of JAK2 in osteoclasts resulted in changes in bone health or osteoclast biology. First, we performed Dual-energy x ray absorptiometry (DXA) scan on excised femurs and lumbar vertebrae (LV). Both male and female *Ctsk*-Cre^+^*Jak2^fl/fl^* mice had reduced femoral BMD at 24 weeks of age compared with littermate controls; however, this difference was no longer significant after adjustment for body length (Figure 3, A and B). Since measurement of areal BMD underestimates bone density in the setting of short stature or growth delay, we applied the principles from the Pediatric Official Positions statement (International society of Clinical Densiometry), which recommend adjusting areal BMD to height (13). At the lumbar spine, there were no significant differences in BMD at LV1–3 both before and after adjusting for body length (Figure 3, C and D).

**Figure 4:**
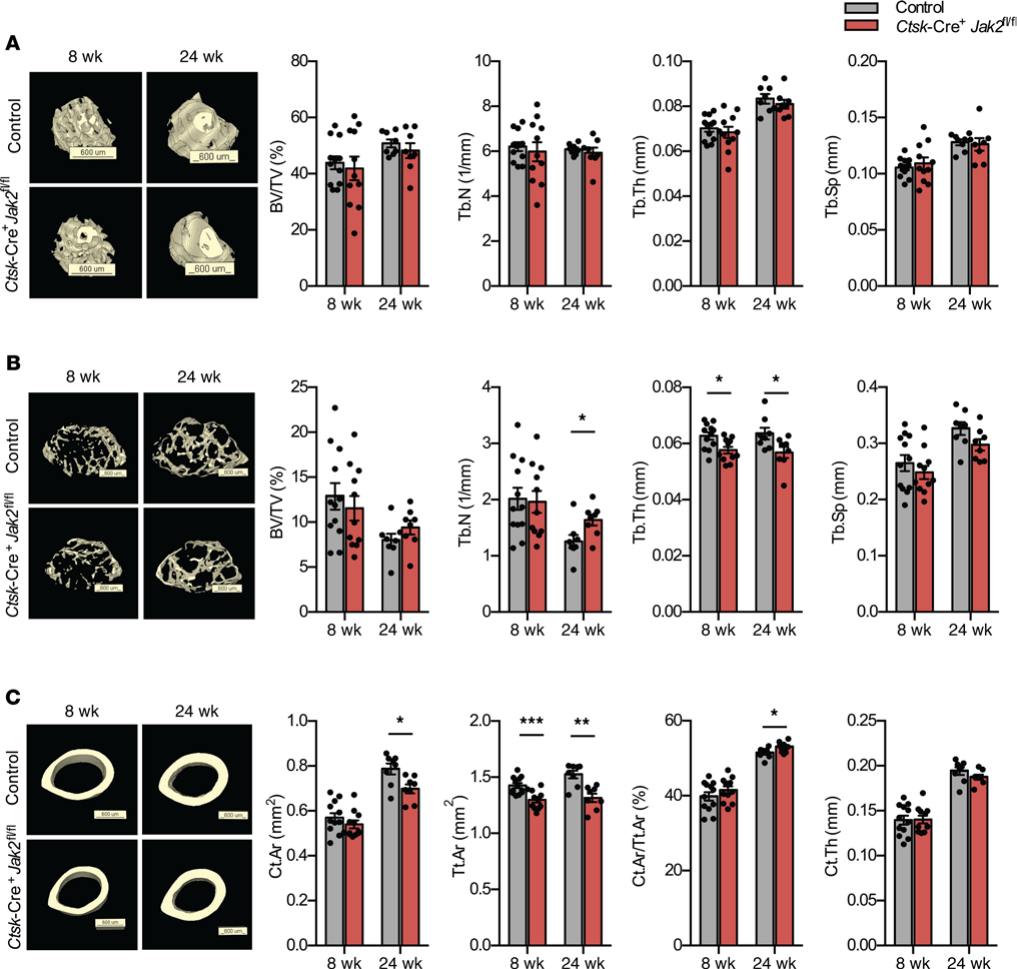
μCT analysis of femurs from *Ctsk*-Cre^+^*Jak2^fl/fl^* mice. Bone microarchitecture was assessed in female *Ctsk*-Cre^+^J*ak2^fl/fl^* and control mice at 8 and 24 weeks of age by ex vivo μCT. (**A**) Three-dimensional reconstruction of the femur neck and quantification of percent bone volume (BV/TV), trabecular number (Tb.N), trabecular thickness (Tb.Th), and trabecular separation (Tb.Sp) in control [*n* = 12 (8 weeks), *n* = 8 (24 weeks)] and *Ctsk*-Cre^+^*Jak2^fl/fl^* [*n* = 11 (8 weeks), *n* = 8 (24 weeks)] mice. (**B**) Three dimensional reconstruction of the distal femur and quantification of BV/TV, Tb.N, Tb.Th, and Tb.Sp in control [*n* = 12 (8 weeks), *n* = 8 (24 weeks)] and *Ctsk*-Cre^+^*Jak2^fl/fl^* [*n* = 11 (8 weeks), *n* = 8 (24 weeks)] mice. (**C**) Three-dimensional reconstruction of the femur midpoint and quantification of cortical area (Ct.Ar), total area (Tt.Ar), percent cortical area to total area (Ct.Ar/Tt.Ar), and cortical thickness (Ct.Th) in control [*n* = 12 (8 weeks), *n* = 8 (24 weeks)] and *Ctsk*-Cre^+^*Jak2^fl/fl^* [*n* = 11 (8 weeks), *n* = 8 (24 weeks)] mice. Data represent mean ± SEM. Differences between groups were analyzed for statistical significance by 2-way ANOVA; *P < 0.05, ***P < 0.001.

Bone microarchitecture of femurs from 8-week-old and 24-week-old female mice was then assessed by μCT. At the femur neck, there were no changes in trabecular bone measures, including bone volume fraction and trabecular number, thickness, or separation (Figure 4A). At the distal femur, *Ctsk*-Cre^+^*Jak2^fl/fl^* mice had reduced trabecular thickness at 8 and 24 weeks, but there were no changes in trabecular number, separation, or overall bone volume fraction (Figure 4B). At the femur midpoint, *Ctsk*-Cre^+^*Jak2^fl/fl^* mice showed reduction in total area and cortical area; however, the cortical area/total area ratio and cortical thickness were unchanged (Figure 4C). Overall, the μCT data show some minor changes in bone microarchitecture, which are likely attributable to the differences in body size of *Ctsk*-Cre^+^*Jak2^fl/fl^* mice.

**Figure 5:**
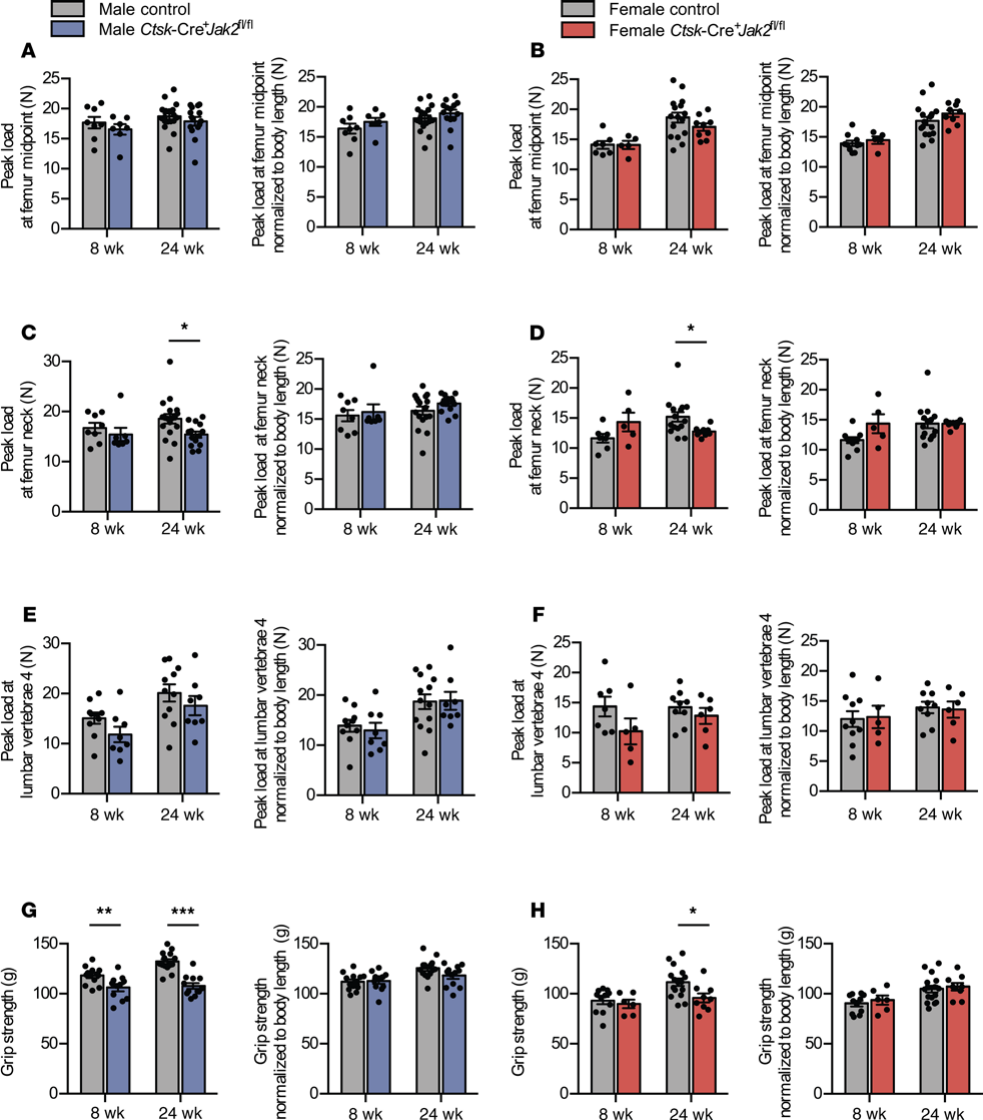
*Ctsk*-Cre^+^*Jak2^fl/fl^* mice have proportional reductions in musculoskeletal strength. Musculoskeletal strength of *Ctsk*-Cre^+^*Jak2^fl/fl^* and control mice was assessed using a materials testing system and grip strength meter. (**A**) Femur midpoint peak load (left panel) and peak load normalized to body length (right panel) in male control [*n* = 8 (8 weeks), *n* = 18 (24 weeks)] and *Ctsk*-Cre^+^*Jak2^fl/fl^* [*n* = 7 (8 weeks), *n* = 14 (24 weeks)] mice. (**B**) Femur midpoint peak load (left panel) and peak load normalized to body length (right panel) in female control [*n* = 7 (8 weeks), *n* = 16 (24 weeks)] and *Ctsk*-Cre^+^*Jak2^fl/fl^* [*n* = 5 (8 weeks), *n* = 9 (24 weeks)] mice. (**C**) Femur neck peak load (left panel) and peak load normalized to body length (right panel) in male control [*n* = 8 (8 weeks), *n* = 18 (24 weeks)] and *Ctsk*-Cre^+^*Jak2^fl/fl^* [*n* = 7 (8 weeks), *n* = 14 (24 weeks)] mice. (**D**) Femur neck peak load (left panel) and peak load normalized to body length (right panel) in female control [*n* = 7 (8 weeks), *n* = 15 (24 weeks)] and *Ctsk*-Cre^+^*Jak2^fl/fl^* [*n* = 5 (8 weeks), *n* = 9 (24 weeks)] mice. (**E**) Lumbar vertebra 4 (LV4) peak load (left panel) and peak load normalized to body length (right panel) in male control [*n* = 10 (8 weeks), *n* = 11 (24 weeks)] and *Ctsk*-Cre^+^*Jak2^fl/fl^* [*n* = 8 (8 weeks), *n* = 8 (24 weeks)] mice. (**F**) LV4 peak load (left panel) and peak load normalized to body length (right panel) in female control [*n* = 7 (8 weeks), *n* = 9 (24 weeks)] and *Ctsk*-Cre^+^*Jak2^fl/fl^* [*n* = 5 (8 weeks), *n* = 6 (24 weeks)] mice. (**G**) Grip strength (left panel) and grip strength normalized to body length (right panel) in male control [*n* = 13 (8 weeks), *n* = 14 (24 weeks)] and *Ctsk*-Cre^+^*Jak2^fl/fl^* [*n* = 11 (8 weeks), *n* = 12 (24 weeks)] mice. (**H**) Grip strength (left panel) and grip strength normalized to body length (right panel) in female control [*n* = 11 (8 weeks), *n* = 16 (24 weeks)] and *Ctsk*-Cre^+^*Jak2^fl/fl^* [*n* = 6 (8 weeks), *n* = 9 (24 weeks)] mice. Data represent mean ± SEM. Differences between groups were analyzed for statistical significance by 2-way ANOVA; *P < 0.05, **P < 0.01, ***P < 0.001.

Next, we assessed biomechanical strength of femurs and LV using a materials testing system. Three-point bending at the femur midpoint showed no difference in peak load both before and after adjustment for body size (Figure 5, A and B). At the femur neck, both male and female *Ctsk*-Cre^+^*Jak2^fl/fl^* mice had reduced compressive peak load at 24 weeks of age; however, this was no longer significant after adjustment for body size (Figure 5, C and D). At the lumbar spine, there was a nonsignificant trend toward reduced compressive peak load in *Ctsk*-Cre^+^*Jak2^fl/fl^* mice, which did not persist after adjustment for body size (Figure 5, E and F). Forelimb grip strength was also measured using a rodent grip strength meter. Male *Ctsk*-Cre^+^*Jak2^fl/fl^* mice had reduced grip strength at 8 and 24 weeks, while female *Ctsk*-Cre^+^*Jak2^fl/fl^* mice demonstrated reduced grip strength at 24 weeks; however, these differences did not persist after adjustment for body size (Figure 5, G and H). Overall, the biomechanical strength testing shows reductions in musculoskeletal strength in *Ctsk*-Cre^+^*Jak2^fl/fl^* mice that are proportional to body size.

**Figure 6:**
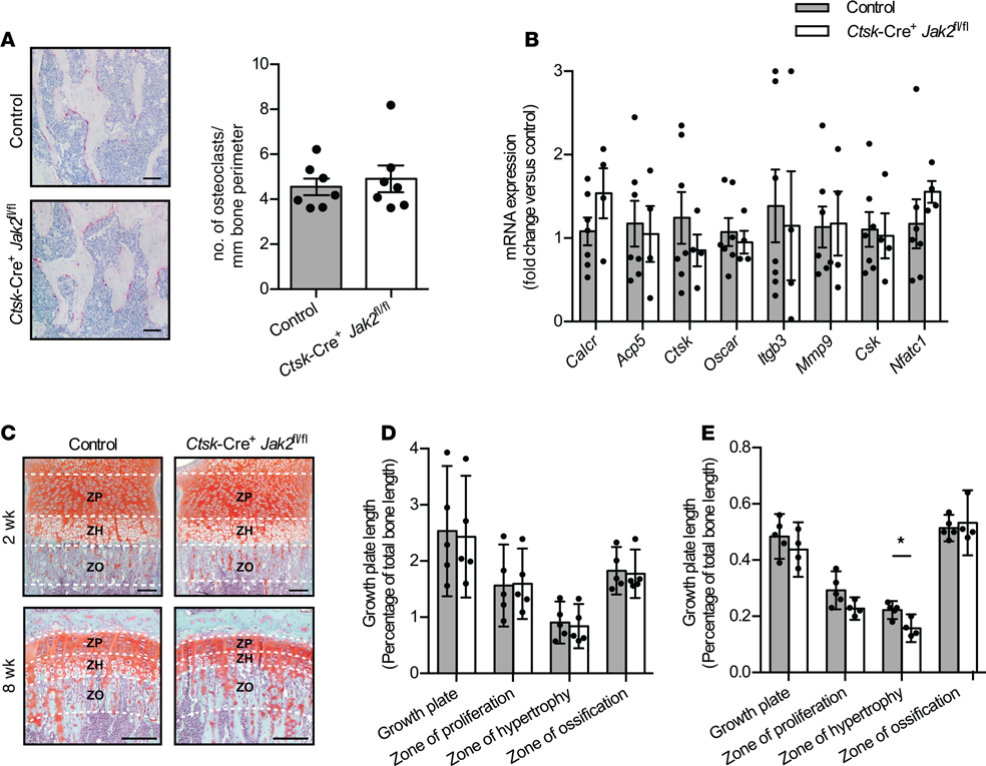
Reduced size of the hypertrophic growth plate zone in *Ctsk*-Cre^+^*Jak2^fl/fl^* mice. (**A**) Representative images of tartrate-resistant alkaline phosphatase–stained (TRAP-stained) sections of distal femurs (left) and quantification of osteoclast number per millimeter of bone perimeter (right) in control (*n* = 7) and *Ctsk*-Cre^+^*Jak2^fl/fl^* mice (*n* = 7). Scale bar: 50 μm. (**B**) mRNA expression of calcitonin receptor (*Calcr*), tartrate-resistant acid phosphatase type 5 (*Acp5*), cathepsin-k (*Ctsk*), osteoclast-associated receptor (*Oscar*), integrin β 3 (*Itgb3*), matrix metalloproteinase 9 (*Mmp9*), C-terminal Src kinase (*Csk*), and nuclear factor of activated t cells 1 (*Nfatc1*) in bone from control (*n* = 7) and *Ctsk*-Cre^+^*Jak2^fl/fl^* mice (*n* = 4). Values are normalized to 18S mRNA levels and presented as fold change over control group. (**C**) Representative images of Safranin O–stained sections of tibia growth plates from 2- and 8-week-old control [*n* = 5 (2 weeks), *n* = 5 (8 weeks)] and *Ctsk*-Cre^+^*Jak2^fl/fl^* [*n* = 5 (2 weeks), *n* = 4 (8 weeks)] mice. ZP, zone of proliferation; ZH, zone of hypertrophy; ZO, zone of ossification; growth plate = ZP + ZH). Scale bars: 100 μm. (**D**) Quantification of tibia growth plate length relative to total bone length in 2-week-old male and female control (*n* = 5) and *Ctsk*-Cre^+^*Jak2^fl/fl^* (*n* = 5) mice. (**E**) Quantification of tibia growth plate length relative to total bone length in 8-week-old male and female control (*n* = 5) and *Ctsk*-Cre^+^*Jak2^fl/fl^* (*n* = 4) mice. Data represent mean ± SEM. Differences between groups were analyzed for statistical significance by Student’s unpaired *t* test; *P < 0.05.

Next, we assessed for effects of JAK2 deficiency on osteoclast parameters. TRAP stain to identify osteoclasts in femur sections showed no alteration in osteoclast numbers or distribution in *Ctsk*-Cre^+^*Jak2^fl/fl^* mice (Figure 6A). Expression of key genes involved in osteoclastogenesis and osteoclast function were quantified in whole bone samples from the distal femur and proximal tibia (Figure 6B). There were no differences in expression of calcitonin receptor (*Calcr*), tartrate-resistant acid phosphatase type 5 (*Acp5*), *Ctsk*, osteoclast-associated receptor (*Oscar*), integrin β 3 (*Itgb3*), matrix metalloproteinase 9 (*Mmp9*), C-terminal Src kinase (*Csk*), and nuclear factor of activated t cells 1 (*Nfatc1*).

Growth plates were then evaluated on Safranin O–stained sections of tibia (Figure 6C). At 2 weeks of age, there were no differences in the size of the growth plate (Figure 6D); however, at 8 weeks of age, *Ctsk*-Cre^+^*Jak2^fl/fl^* mice demonstrated a significant reduction in the size of the zone of hypertrophy (ZH) compared with control mice but not in the size of the total growth plate, zone of proliferation (ZP), or zone of ossification (ZO) (Figure 6E).

Overall, these data show that deletion of JAK2 in osteoclasts does not have a positive or negative effect on bone density, bone microarchitecture, biomechanical strength, osteoclast number, or expression of osteoclastogenic genes. Histologically, there was a reduction in the size of the ZH in *Ctsk*-Cre^+^*Jak2^fl/fl^* mice in association with their attenuation in body growth.

### Reduced osteoclast-specific expression of IGF1 in Ctsk-Cre^+^Jak2^fl/fl^ mice.

Growth defects in osteoclast-specific KO mice often arise due to impaired osteoclast function and an inability to resorb bone at the developing growth plate (14–15). Despite clear growth defects in *Ctsk*-Cre^+^*Jak2^fl/fl^* mice, there was surprisingly no evidence of defects in osteoclast function. Thus, we sought to investigate an alternative mechanism to explain the growth defects observed in *Ctsk*-Cre^+^*Jak2^fl/fl^* mice.

**Figure 7:**
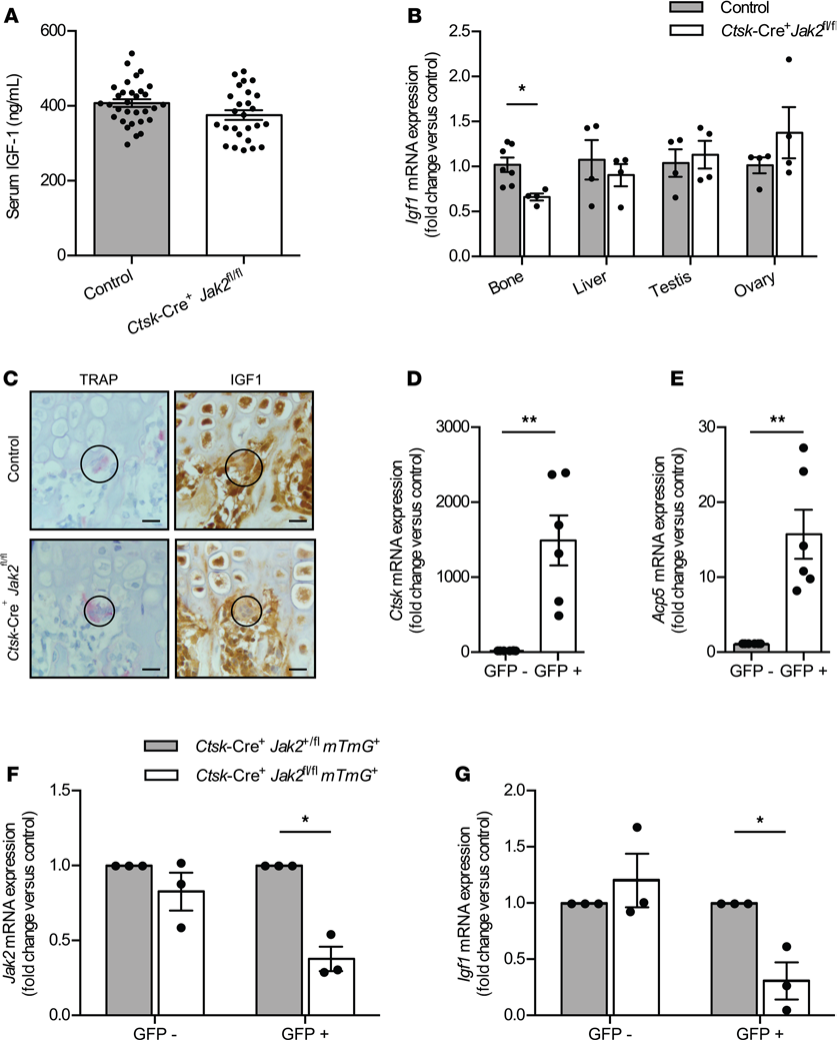
Reduced osteoclast-specific expression of IGF1 in *Ctsk*-Cre^+^*Jak2^fl/fl^* mice. Expression of IGF1 was assessed in tissues and osteoclasts from 8-week-old *Ctsk*-Cre^+^*Jak2^fl/fl^* and control mice. (**A**) Serum IGF1 concentrations in male and female control (*n* = 31) and *Ctsk*-Cre^+^*Jak2^fl/fl^* (*n* = 26) mice at 8 weeks of age. (**B**) mRNA expression of *Igf1* in bone (distal femur and proximal tibia), liver, testis, and ovary from control (*n* = 4, [*n* = 7 for bone]) and *Ctsk*-Cre^+^*Jak2^fl/fl^* mice (*n* = 4) mice. Values are normalized to *18S* mRNA levels and presented as fold change over control group. (**C**) Representative images of femur from control (*n* = 3) and *Ctsk*-Cre^+^*Jak2^fl/fl^* mice (*n* = 3) stained for TRAP and immunostained for IGF1. Staining was performed on adjacent serial sections. The circles highlight TRAP^+^ multinucleated giant cells. Scale bars: 10 μm. (**D**) mRNA expression of *Ctsk* in FACS GFP^–^ and GFP^+^ bone cells from *Ctsk*-Cre^+^*Jak2^fl/+^mTmG*^+^ (*n* = 3) and *Ctsk*-Cre^+^*Jak2^fl/fl^mTmG*^+^ mice (*n* = 3). Values are normalized to *18S* mRNA levels and presented as fold change over GFP^–^ cells. (**E**) mRNA expression of *Acp5* in FACS GFP^–^ and GFP^+^ bone cells from *Ctsk*-Cre^+^*Jak2^fl/+^mTmG*^+^ (*n* = 3) and *Ctsk*-Cre^+^*Jak2^fl/fl^mTmG*^+^ mice (*n* = 3). Values are normalized to *18S* mRNA levels and presented as fold change over GFP^–^ cells. (**F**) mRNA expression of *Jak2* in FACS GFP^–^ and GFP^+^ bone cells from *Ctsk*-Cre^+^*Jak2^fl/+^mTmG*^+^ (*n* = 3) and *Ctsk*-Cre^+^*Jak2^fl/fl^mTmG*^+^ mice (*n* = 3). Values are normalized to *18S* mRNA levels and presented as fold change over control group. (**G**) mRNA expression of *Igf1* in FACS GFP^–^ and GFP^+^ bone cells from *Ctsk*-Cre^+^*Jak2^fl/+^mTmG*^+^ (*n* = 3) and *Ctsk*-Cre^+^*Jak2^fl/fl^mTmG*^+^ mice (*n* = 3). Values are normalized to *18S* mRNA levels and presented as fold change over control group. Data represent mean ± SEM. Differences between groups were analyzed for statistical significance by Student’s unpaired *t* test; *P < 0.05, **P < 0.01.

Since Oc-JAK2–KO led to overall proportional changes in body size and reductions in the growth plate ZH, we hypothesized that a secreted growth factor might play an important role. JAK2 is a well-established regulator of IGF1 expression (16–19); therefore, we assessed IGF1 levels in *Ctsk*-Cre^+^*Jak2^fl/fl^* mice. Serum IGF1 concentrations, measured by ELISA, were equivalent in control and *Ctsk*-Cre^+^*Jak2^fl/fl^* mice at 8 weeks of age (Figure 7A). Quantitative PCR (qPCR), however, showed a decrease in *Igf1* mRNA expression in the distal femur/proximal tibia in *Ctsk*-Cre^+^*Jak2^fl/fl^* compared with controls, while no changes in *Igf1* expression in other tissues — including the liver, testis, and ovary — were present (Figure 7B). To determine if the decreased expression of IGF1 in bone was specifically from osteoclasts, we first performed IHC for IGF1 in femur sections (Figure 7C). In both control and KO mice, there was diffuse staining for IGF1 in BM cells and chondrocytes, as previously reported (20). In TRAP^+^ multinucleated giant cells, immunostaining for IGF1 was of lower intensity in Ctsk-Cre^+^Jak2*^fl/fl^* mice compared with controls, while the surrounding BM cells were unaffected. To further confirm a reduction of osteoclast-specific IGF1, we measured *Igf1* gene expression in osteoclasts isolated by FACS. We used control and Ctsk-Cre^+^Jak2*^fl/fl^* mice bred to the *mTmG* reporter mouse and isolated the GFP^+^ bone cells. Compared with GFP^–^ cells, purified GFP^+^ cells had a marked increase in expression of *Ctsk* (Figure 7D) and *Acp5* (Figure 7E), consistent with these being mature osteoclasts. *Jak2* mRNA was reduced by 62% in GFP^+^ cells in *Ctsk*-Cre^+^*Jak2^fl/fl^mTmG*^+^ mice compared with osteoclast *Jak2* heterozygous (*Ctsk*-Cre^+^*Jak2^fl/+^* *mTmG*^+^) control mice (Figure 7F). Similarly, *Igf1* mRNA was reduced by approximately 70% in GFP^+^ cells from *Ctsk*-Cre^+^*Jak2^fl/fl^mTmG*^+^ mice compared with osteoclast *Jak2* heterozygous mice (Figure 7G). No difference in either *Jak2* or *Igf1* expression was detected in GFP^–^ cells. Overall, these findings are consistent with reduced osteoclast-specific IGF1 expression in *Ctsk*-Cre^+^*Jak2^fl/fl^* mice.

### Ctsk-Cre^+^Igf1^fl/fl^ mice demonstrate postnatal growth restriction.

**Figure 8:**
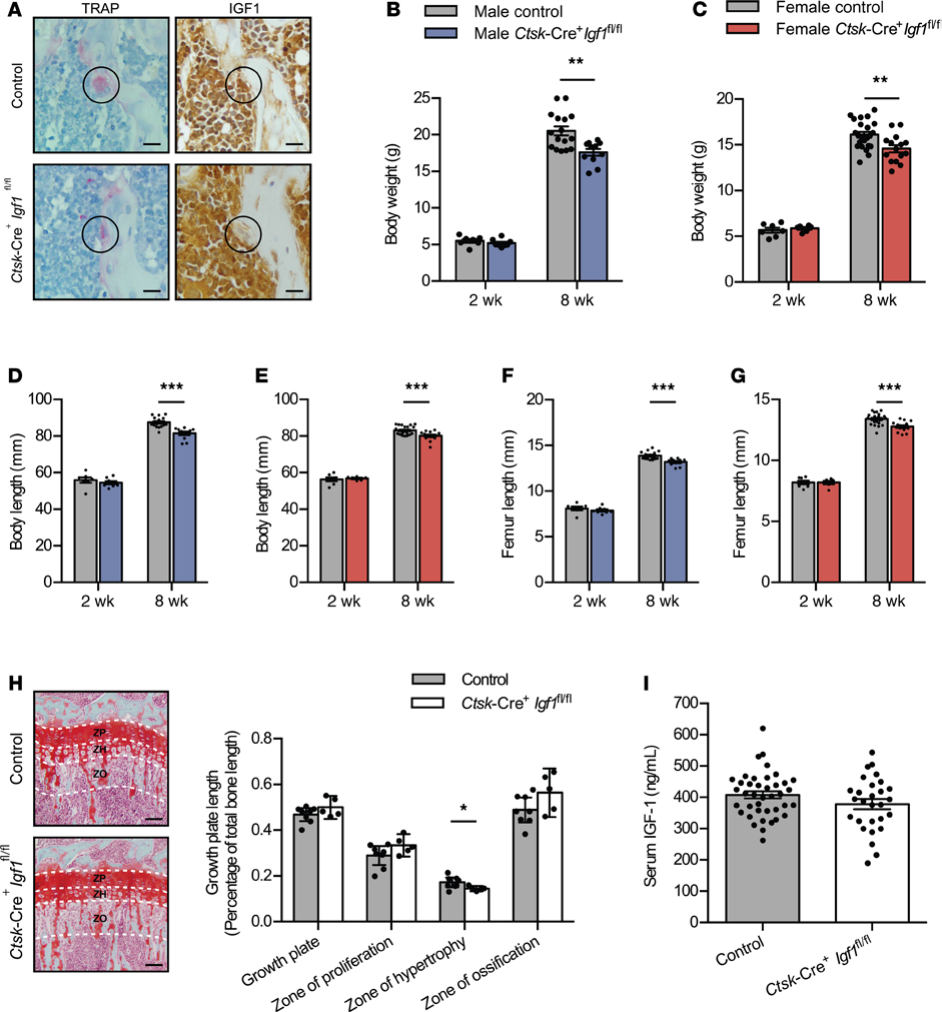
*Ctsk*-Cre^+^*Igf1^fl/fl^* mice demonstrate postnatal growth restriction. *Ctsk*-Cre^+^*Igf1^fl/fl^* and littermate control mice were followed until 8 weeks of age, and growth parameters were assessed. (**A**) Representative images of femur from control (*n* = 3) and *Ctsk*-Cre^+^*Igf1^fl/fl^* mice (*n* = 3) stained for TRAP and immunostained for IGF1. Staining was performed on adjacent serial sections. The circles highlight TRAP^+^ multinucleated giant cells. Scale bars: 10 μm. (**B**) Body weight of male control and *Ctsk*-Cre^+^*Igf1^fl/fl^* mice. (**C**) Body weight of female control and *Ctsk*-Cre^+^*Igf1^fl/fl^* mice. (**D**) Body length of male control and *Ctsk*-Cre^+^*Igf1^fl/fl^* mice. (**E**) Body length of female control and *Ctsk*-Cre^+^*Igf1^fl/fl^* mice. (**F**) Femur length of male control and *Ctsk*-Cre^+^*Igf1^fl/fl^* mice. (**G**) Femur length of female control and *Ctsk*-Cre^+^*Igf1^fl/fl^* mice. Control males [*n* = 7 (2 weeks), *n* = 16 (8 weeks)] and *Ctsk*-Cre^+^*Igf1^fl/fl^* males [*n* = 8 (2 weeks), *n* = 11 (8 weeks)] mice. Control females [*n* = 7 (2 weeks), *n* = 23 (8 weeks)] and *Ctsk*-Cre^+^*Igf1^fl/fl^* females [*n* = 6 (2 weeks), *n* = 14 (8 weeks)]. (**H**) Representative photographs of Safranin O–stained tibia growth plates and quantification of tibia growth plate length relative to total bone length in 8-week-old control (*n* = 8) and *Ctsk*-Cre^+^*Igf1^fl/fl^* (*n* = 5) mice (ZP, zone of proliferation; ZH, zone of hypertrophy; ZO, zone of ossification; growth plate = ZP + ZH). Scale bars: 100 μm. (**I**) Serum IGF1 concentrations in male and female control (*n* = 40) and *Ctsk*-Cre^+^*Igf1^fl/fl^* (*n* = 28) mice at 8 weeks of age. Data represent mean ± SEM. Differences between groups were analyzed for statistical significance by 2-way ANOVA or Student’s unpaired *t* test; *P < 0.05, **P < 0.01, ***P < 0.001.

Given our results showing growth defects with reduced osteoclast-derived IGF1 in *Ctsk*-Cre^+^*Jak2^fl/fl^* mice, we hypothesized that a causal link exists between osteoclast-derived IGF1 and postnatal growth. To test this hypothesis, we generated osteoclast-specific IGF1–KO mice (*Ctsk*-Cre^+^*Igf1^fl/fl^* mice). IHC performed on femur sections confirmed loss of IGF1 specifically in TRAP^+^ multinucleated giant cells in *Ctsk*-Cre^+^*Igf1^fl/fl^* mice (Figure 8A). Similar to *Ctsk*-Cre^+^*Jak2^fl/fl^* mice, both male and female *Ctsk*-Cre^+^*Igf1^fl/fl^* mice had small but statistically significant reductions in body weight (Figure 8, B and C), body length (Figure 8, D and E), and femur length (Figure 8, F and G) compared with littermate controls at 8 weeks of age, but not at 2 weeks. The extent of reduction in growth parameters was also similar between the 2 gene KO models. Additionally, histological analysis of the growth plate showed a decrease in the size of the ZH without changes in the ZO (Figure 8H). Serum IGF1 concentrations were similar in control and *Ctsk*-Cre^+^*Igf1^fl/fl^* mice (Figure 8I). Overall, these findings parallel those of *Ctsk*-Cre^+^*Jak2^fl/fl^* mice and support the hypothesis that osteoclast-derived IGF1 regulates postnatal growth.

### Transgenic overexpression of IGF1 rescues growth defects in Ctsk-Cre^+^Jak2^fl/fl^ mice.

**Figure 9:**
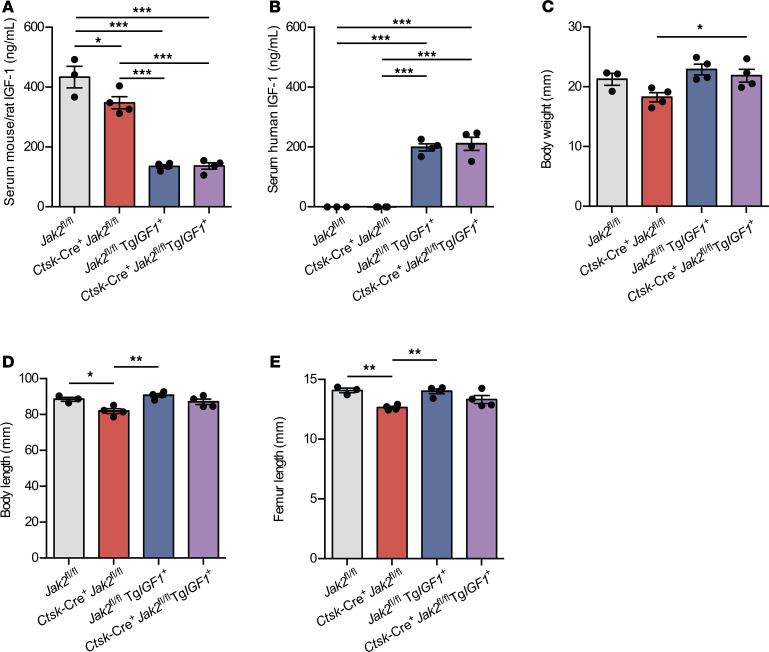
Transgenic overexpression of IGF1 rescues growth defects in *Ctsk*-Cre^+^*Jak2^fl/fl^* mice. *Ctsk*-Cre^+^*Jak2^fl/fl^* mice were bred to a transgenic mouse overexpressing human IGF1 (Tg*IGF1*^+^), and growth parameters of male offspring were assessed at 8 weeks of age. (**A**–**E**) Serum mouse/rat IGF1 concentrations (**A**), serum human IGF1 concentrations (**B**), body weight (**C**), body length (**D**), and femur length (**E**) of *Jak2^fl/fl^* (*n* = 3), *Ctsk*-Cre^+^*Jak2^fl/fl^* (*n* = 4), *Jak2^fl/fl^*Tg*IGF1*^+^ (*n* = 4), and *Ctsk*-Cre^+^*Jak2^fl/fl^*Tg*IGF1*^+^ (*n* = 4) mice. Data represent mean ± SEM. Differences between groups were analyzed for statistical significance by 1-way ANOVA; *P < 0.05, **P < 0.01, ***P < 0.001.

Since osteoclast expression of IGF1 was found to play a role in body size determination, we sought to determine if the decreased expression of IGF1 in *Ctsk*-Cre^+^*Jak2^fl/fl^* mice had a causal role in their attenuated growth. To this end, we used a transgenic IGF1–overexpressing mouse, which expresses human *IGF1* ([h]*IGF1*) under control of the transthyretin (*Ttr*) promotor to increase circulating IGF1. This model has previously been shown to overcome both endocrine and local deficits in IGF1 in order to restore normal growth (20). Analysis of serum showed equivalent reductions in circulating mouse IGF1 in both control and *Ctsk*-Cre^+^*Jak2^fl/fl^* mice carrying the (h)*IGF1* transgene (Figure 9A). Moreover, levels of human IGF1 in circulation were also similar between these mice (Figure 9B). Mouse IGF1 was likely reduced in the presence of the (h)*IGF1* transgene due to feedback inhibition of endogenous IGF1. (h)*IGF1* expression did not alter growth parameters in control mice. This is consistent with previous studies showing no effect on body length and femur length in (h)*IGF1*-expressing mice (21). In *Ctsk*-Cre^+^*Jak2^fl/fl^* mice, however, transgenic expression of (h)*IGF1* resulted in normalization of body weight (Figure 9C), body length (Figure 9D), and femur length (Figure 9E) at 8 weeks of age. These findings suggest a causal role between reduced IGF1 expression in *Ctsk*-Cre^+^*Jak2^fl/fl^* mice and postnatal growth restriction.

## Discussion

In this study, we investigated the in vivo role of JAK2 in osteoclasts using Cre/Lox-P–mediated gene deletion. Although Oc-JAK2–KO mice demonstrated postnatal growth restriction, we did not identify any structural or functional alterations in bone, and any changes observed were proportional to the change in body size, suggesting that JAK2 is not essential for canonical bone resorptive function in osteoclasts. We assessed multiple measures of bone homeostasis in both sexes and at multiple time points. Since peak trabecular bone mass is achieved by 8 weeks and then declines (22), our data show that osteoclast JAK2 is dispensable for both normal bone development and in modulating age-related bone loss.

Previous studies have hinted at a potential role for JAK2 in osteoclast biology, since cytokines and growth factors that signal through JAK2 have been reported to modulate osteoclastogenesis (23). For example, growth hormone has been reported to promote osteoclastogenesis from unfractionated bone cells through direct effects on osteoclasts and indirectly through effects on stromal cells (24). IL-6 has been shown to inhibit osteoclastogenesis in BM macrophages and RAW 264.6 cell, which is dependent on STAT3 activation (25). IL-12, which also signals through JAK2-mediated STAT phosphorylation, inhibits osteoclastogenesis indirectly through induction of IFN-γ in stromal and immune cells (26). RANKL, which is essential for osteoclast formation, is also dependent on JAK-STAT signaling (27). RANKL-stimulated osteoclast formation is associated with reduced protein expression of JAK1, which prevents IFN-β inhibition of osteoclast lineage commitment. However, there are no alterations in JAK2 or JAK3 levels upon RANKL stimulation, suggesting that JAK2 is not essential, whereas JAK1 may be essential in the differentiation of osteoclasts. In terms of studies investigating JAK2 specifically, the JAK2 inhibitor AG490 was shown to promote osteoclast survival in vitro upon RANKL withdrawal (11). There is, however, doubt about the specificity of AG490 for JAK2, since it has been reported to also inhibit JAK3, STAT1/3/5, activating protein 1, and mitogen-activated protein kinase pathways; thus, it is unclear whether the effects observed in this study can be attributed specifically to inhibition of JAK2 (28). Overall, the studies linking JAK2-mediated signaling with osteoclast differentiation and survival are limited by the fact that osteoclast biology has only been investigated in vitro and that many of the effects seen may be due to indirect effects on nonosteoclast cells. Based on the results from our current study, we find that JAK2 does not have an appreciable effect on osteoclast formation or resorptive function in vivo. Further investigation, however, may be needed to determine if there is a role of osteoclast JAK2 in the setting of advanced age or sex hormone deficiency.

Despite the lack of bone resorptive effects seen in this study, Oc-JAK2–KO mice displayed significant postnatal growth restriction. Many in vivo gene KO studies have been performed in osteoclasts resulting in growth defects, which often arise in conjunction with impaired osteoclast function in vivo resulting in osteopetrosis (14–15). This is accompanied by an increase in bone mass and other defects, including doming of the skull, delayed tooth eruption, and radio densities within regions of the BM. In our study, Oc-JAK2–KO mice did not display an increase in bone mass or any stigmata of osteopetrosis; therefore, it is unlikely that growth restriction occurred as a result of impairment in the traditional osteoclast function in bone resorption. We therefore propose a potentially novel role for osteoclasts in determining body growth independently of their canonical function in bone mineral homeostasis. Since JAK2 is a well-established regulator of IGF1 expression, most classically from the liver (16–19), we hypothesized that JAK2 signaling in osteoclasts might similarly regulate IGF1 expression. The body growth restriction observed in Oc-JAK2–KO mice was associated with reduced expression of IGF1 in bone and osteoclasts. Overexpression of IGF1 abolished these growth defects, suggesting a causal role between JAK2-mediated IGF1 expression in osteoclasts and determination of body size. Additionally, when IGF1 was directly targeted in osteoclasts, this produced almost identical phenotypic findings compared with Oc-JAK2–KO mice. Together, these data support a role for osteoclast-derived IGF1 as a small but significant contributor to postnatal growth.

IGF1 is a ubiquitously expressed hormone and plays an essential role in postnatal growth (29). It is well established that approximately 75% of circulating IGF1 is derived from the liver (30–31). Our understanding, however, of extrahepatic sources of IGF1 remains unclear, and there is an emerging recognition of the importance of autocrine and paracrine functions of IGF1. For example, targeted deletion of IGF1 in chondrocytes (32), osteoblasts (33), or osteocytes (34) produces unique effects on bone homeostasis and growth, without affecting circulating IGF concentrations. Our study provides evidence that IGF1 in osteoclasts similarly plays an important role in postnatal growth. Given that we saw changes in IGF1 in osteoclasts and bone, but not in circulating IGF1 concentrations, this would support a role for local autocrine or paracrine actions of osteoclast-derived IGF1. It has previously been shown that IGF1 is an important stimulator of chondrocyte hypertrophy (35), and in our study, a reduction in the hypertrophic zone was the only histological change seen in the growth plate. Thus, it is reasonable to suspect that osteoclast-derived IGF1 might act locally on chondrocytes in this zone, since they are located immediately adjacent to the ZO, where numerous active osteoclasts are located. We found the ZH to be reduced in size without associated changes in the overall size of the growth plate. This may be due to inadequate sample size or missing growth plate changes at other critical time points during this dynamic process. Overall, further research is needed to delineate the specific local actions of osteoclast-derived IGF1; however, the details of this are beyond the scope of our current study.

In conclusion, this study describes a potentially novel role for Oc-JAK2 and IGF1 in the regulation of postnatal growth and determination of body size. Apart from the role of Oc-JAK2 in body growth, we show that under physiologic conditions, Oc-JAK2 appears to be dispensable for the canonical function of osteoclasts and not essential to the development and homeostasis of bone mass, strength, or microarchitecture in vivo.

## Methods

### Animals.

*Ctsk*-Cre mice (*Ctsk*^tm1[cre]Ska^) (36) were obtained from Wentian Yang (Brown University Alpert Medical School, Providence, Rhode Island, USA). *Jak2^fl/fl^* mice (*Jak2*^tm1Kuw^) (37) were obtained from Kay-Uwe Wagner (Wayne State University, Detroit, Michigan, USA). *mTmG* reporter mice (Gt[ROSA]26Sor^tm4[ACTB–tdTomato,–EGFP]Luo^/J, stock no. 007576) (38), *Igf1* floxed mice (B6.129[FVB]-*Igf1*^tm1Dlr^/J, stock no. 016831) (39), and transgenic IGF1–overexpressing mice (FVB-Tg[*Ttr-IGF1*]1Sykr/J, stock no. 012662) (21) were obtained from the Jackson Laboratory. Oc-JAK2–KO mice were generated by breeding heterozygous *Ctsk*-Cre*^+/–^* mice to *Jak2^fl/fl^* mice to generate *Ctsk*-Cre*^+/–^Jak2^fl/+^*mice. *Ctsk*-Cre*^+/–^Jak2^fl/+^*mice were back crossed to *Jak2^fl/fl^* mice to generate Oc-JAK2–KO mice (*Ctsk*-Cre*^+/–^Jak2^fl/fl^*) and littermate control mice (*Jak2^fl/fl^* and *Ctsk*-Cre*^+/–^Jak2^fl/+^*). Both *Jak2^fl/fl^* and *Ctsk*-Cre*^+/–^Jak2^fl/+^* controls were used for all experiments unless otherwise specified. There were no phenotypic differences in any outcomes between *Jak2^fl/fl^* and *Ctsk*-Cre*^+/–^Jak2^fl/+^* mice. Osteoclast-specific IGF1–KO mice (*Ctsk*-Cre*^+^Igf1^fl/fl^*) were generated using the same breeding scheme. For reporter mouse experiments, *Ctsk*-Cre*^+/–^* mice were bred to *mTmG^+/–^* mice to generate *Ctsk*-Cre*^+/–^mTmG^+/–^* mice. For transgenic IGF1 rescue experiments, *Ctsk*-Cre*^+/–^Jak2^fl/fl^* mice were bred to *Jak2^fl/fl^*Tg*IGF1^+/–^* to generate control (*Jak2^fl/fl^*), KO (*Ctsk*-Cre*^+/–^Jak2^fl/fl^*), control with IGF1 overexpression (*Jak2^fl/fl^*Tg*IGF1^+/–^*), and KO with IGF1 overexpression (*Ctsk*-Cre*^+/–^Jak2^fl/fl^* Tg*IGF1^+/–^*) mice. Mice were housed in a pathogen-free facility with a 12-hour light–dark cycle and free access to water and standard rodent chow (5% energy from fat; Harlan Teklad). Body weights were measured weekly beginning at 2 weeks of age. Measurements of body length and bone length were taken at the time of sacrifice using a digital calliper.

### DXA and μCT.

BMD of excised femurs and LV1–3 was measured by DXA (Orthometrix) using a specialized software program (Host Software version 3.9.4; Scanner Software version 1.2.0, Orthometrix). Calibration of the DXA was performed daily using quality control and quality assurance standards. Femurs and LV1–3 were scanned using a resolution of 0.1 mm × 0.1 mm and at a speed of 2 mm/s.

For assessment of bone microarchitecture, left femurs were scanned ex vivo using μCT (Skyscan 1176, Bruker microCT). The bones were wrapped in parafilm to retain moisture during scanning and placed axially in a foam holder for scanning using a 9 μm isotropic voxel size, 0.25 mm aluminum filter, voltage of 45 kV, tube current of 545 μA, 850 ms exposure time, and 0.2 degree rotation step. Scans were performed over 180° without using frame averaging. To reconstruct scanned images, Graphics Processing Unit–acceleration (GPU-acceleration) (GPUReconServer, Skyscan, Bruker microCT) and NRecon Reconstruction 64-bit software (Skyscan, Bruker microCT) were used. Except for variable misalignment compensations, all scanned samples within each skeletal site received the same corrections to smoothing, ring artifacts, beam hardening, and defect pixel masking. Reconstructed images were then reoriented (DataViewer software, version 1.5.0, Skyscan, Bruker microCT), and the transaxial images were saved. Regions of interest (ROIs) were selected from transaxial images and saved as new data sets using CTAnalyzer software (Skyscan Bruker microCT). At the femur neck, an ROI of the trabecular region was manually drawn to a few pixels away from the endocortical boundary and consisted of 1.680 mm (191 slices), starting at the distal edge of the femur head and extending toward the diaphysis of the femur. At the distal femur, the ROI of the trabecular region was manually drawn a few pixels away from the endocortical boundary and consisted of 0.879 mm (100 slices), starting 0.616 mm (70 slices) proximal to the metaphyseal edge of the growth plate and extending toward the femur neck. Another ROI was manually drawn around the midpoint region of the femur, rich in cortical bone, and consisted of 0.879 mm (100 slices) spanning above and below the femur midpoint. Local adaptive thresholding was used to segment the trabecular bone from the background (femur neck: lower threshold = 74, upper threshold = 255, radius = 6, constant = 0; distal femur: lower threshold = 71, upper threshold = 255, radius = 6, constant = 0) and global thresholding (femur: lower threshold = 124, upper threshold = 255) for segmenting the cortical bone from the background at the femur midpoint. At the femur neck and distal femur, the trabecular outcomes determined were bone volume fraction (BV/TV), trabecular number (Tb.N), trabecular thickness (Tb.Th), and trabecular separation (Tb.Sp). Cortical bone outcomes determined at the femur midpoint were total cross-sectional area inside the periosteal envelope (Tt.Ar), cortical bone area (Ct.Ar), cortical area fraction (Ct.Ar/Tt.Ar), and average cortical thickness (Ct.Th).

### Biomechanical strength testing.

Bone strength was measured using a Materials Testing System (Model 4442, Instron Corp.) and specialized software (Bluehill 2, Instron Corp.). To assess bone strength at skeletal sites rich in cortical bone, the peak load at the femur midpoint was determined by 3-point bending. To assess skeletal sites rich in trabecular bone, the maximum compressive force at the femur neck and LV4 was measured. Grip strength was measured using a mouse/rat grip strength meter (Model BIO-GS3, Bioseb). Forelimb grip strength was measured according to the manufacturer’s instructions. Ten consecutive grip strength tests were performed, and the best 3 attempts were averaged to give maximal grip strength.

### Histology.

Femurs and tibia were cleaned and fixed in 10% neutral buffered formalin for > 48 hours and decalcified in 14% EDTA (pH 7.2) for 2 weeks, and they were then paraffin embedded and cut into 5 μm sagittal sections. For TRAP staining, rehydrated sections were incubated in TRAP staining solution (sodium acetate [0.11M], L-tartaric acid [76.0 mM], napthol AS-MX phosphate [0.27M], ethylene glycol monoethyl ether [0.5% v/v], and Fast Red Violet LB Salt [1.59M]; pH 4.5] for 30 minutes at 37°C, counterstained in fast green solution (0.08% fast green, 1% acetic acid) or hematoxylin, and then air-dried, dipped in xylene and mounted. For Safranin O staining, rehydrated slides were incubated in Mayer’s Hematoxylin No.2 for 10 minutes, Safranin O (0.1%) for 5 minutes, and fast green solution for 1 minute and then air-dried, dipped in xylene, and mounted. For IHC, samples were collected after perfusion fixation with 10% neutral buffered formalin before being fixed for 24 hours and decalcified in 10% EDTA (pH 7.4) for 10 days. Samples were paraffin embedded and cut into 4 μm sections. Heat-mediated antigen retrieval was performed in 10 mM sodium citrate (pH 6) at 60°C for 16 hours, followed by blocking in PBS with 1% BSA and 10% goat serum for 30 minutes. Sections were incubated with rabbit primary antibody diluted 1:50 for 3 hours at room temperature (JAK2, Cell Signaling Technologies, 3230; IGF1, Abcam, 63926) followed by incubation with goat anti–rabbit HRP polymer–linked secondary antibody (SignalStain Boost IHC Detection Reagent, Cell Signaling Technologies, 8114) for 30 minutes. Peroxidase detection was performed using the SignalStain DAB Substrate Kit (Cell Signaling Technologies, 8059) for 10 minutes, followed by counterstaining in hematoxylin. Staining controls with omission of primary antibody were performed, and no immunoreactivity was detected. For fluorescence imaging of reporter mice, euthanized mice were perfused with ice-cold 10% neutral buffered formalin; then, dissected tissues were further fixed for another 3 hours before being cryopreserved in PBS with 15% sucrose for 1 hour and PBS with 15% sucrose and 50% OCT for 3 hours. They were then embedded in OCT medium. Knee joints from 1-week-old mice were embedded without decalcification. Frozen sections (7 μm) were cut, washed in PBS, and mounted in fluroshield mounting medium with DAPI. Immunofluorescence images were obtained by a Zeiss inverted fluorescence microscope (Advanced Optical Microscopy Facility). For determination of growth plate size, histological sections were taken from the midsagittal plane of the proximal tibia, stained with Safranin O, and imaged with a light microscope. The ZP was defined as the area with columns of chondrocytes with a flattened appearance. The ZH was defined as the area where chondrocytes are enlarged, swollen, or vacuolated. The ZO was defined as the area with longitudinal spicules of cartilage surrounded by bone. As depicted in Figure 6C, smooth lines were drawn to delineate each zone by an operator who was blinded to the study groups. Measurements of the length of each zone, in the longitudinal plane of the bone, were made at the midpoint of each line using ImageJ software (NIH).

### Cell culture.

BM was collected from femurs and tibias of 4-week-old mice and suspended in α-MEM media (Thermo Fisher Scientific) supplemented with 10% FBS (Sigma-Aldrich) and 100 units/mL penicillin/streptomycin (Thermo Fisher Scientific), passed through a 40 μm cell strainer and treated with RBC lysis buffer containing ammonium chloride (Cell Signaling Technologies, 46232). BM cells were plated overnight in α-MEM in a T175 flask. Nonadherent cells were collected, and 2.5 × 10^5^ cells were plated in each well of a 24-well plate in α-MEM supplemented with 100 ng/mL murine macrophage CSF (M-CSF; PeproTech, 315-02) for 3 days. For generation of BM-derived osteoclasts, cells were further incubated in α-MEM with 100 ng/mL M-CSF and 100 ng/mL recombinant murine soluble receptor activator of NF-κB ligand (RANKL; PeproTech, 315-11C) for an additional 4 days (media changed on days 2 and 3), and the presence of multinucleated giant cells was confirmed by light microscopy. For generation of BM-derived macrophages, RANKL was omitted from cell cultures.

### qPCR.

Total RNA from cells and tissues was isolated using Trizol reagent (Invitrogen). For isolation of RNA from bone, femurs and tibias were cleaned of soft tissue, and BM was removed by centrifugation (16,000*g* for 10 seconds at 4°C). The distal third of the femur and proximal third of the tibia were then placed in foil and pulverized under liquid nitrogen using a mortar and pestle. The samples were further homogenized in Trizol using Precellys Minilys Personal Tissue Homogenizer (Cayman Chemical). RNA was reverse transcribed with random primers using M-MLV enzyme (Invitrogen). qPCR was performed using specific primers and SYBR Green master mix on a 7900HT Fast-Real-Time PCR System (Applied Biosystems). The relative mRNA abundance of each gene was normalized to the expression level of *18S*. *18S* expression was not significantly different between groups. Primer sequences are listed in Table 2.

### Analysis of serum.

Blood was collected at sacrifice by cardiac puncture. Serum IGF1 was measured using a mouse/rat IGF1 ELISA kit (R&D Systems, MG100) or human IGF1 ELISA kit (R&D Systems, DG100B) as per the manufacturer’s instructions.

### FACS.

Control and KO mice were bred to the *mTmG* reporter mouse to generate mice with GFP^+^ osteoclasts. Single-cell suspensions from long bones were isolated as previously described (40). Briefly, long bones were cleaned of soft tissue and flushed of marrow; they were then cut into 1 mm pieces. The fragments were digested twice for 30 minutes each in HBSS supplemented with 10 mg/mL type IV collagenase (Sigma-Aldrich, C5138) at 37°C with agitation. The solution was passed through a 70 μM cell strainer to remove cell debris/clumps. Cell viability was verified by trypan blue staining and cells were suspended in FACS buffer at a concentration of 10 M/mL. Osteoclasts were sorted based on the expression of GFP through the BD FACSAria-III Digital Cell Sorter (University Health Network Flow Cytometry Facility).

### Statistics.

For comparison of data between 2 groups, 2-tailed independent Student’s *t* test was performed. For comparison among multiple groups, 1-way ANOVA was performed. For comparison between 2 groups at different time points, 2-way ANOVA was performed. For comparison of weekly body weight between 2 groups, repeated-measures 1- or 2-way ANOVA was performed. Post hoc multiple comparisons were performed using the Holm-Sidak method. For normalized data, the residual method was employed where unstandardized residuals were calculated from linear regression and added to a constant. Data are presented as mean ± SEM. Data were analyzed with SPSS version 20 (SPSS Inc.) and Prism GraphPad version 6. Statistical significance was defined as *P* < 0.05.

### Study approval.

All experiments using mice in this study were approved by the Toronto General Hospital Research Institute Animal Care Committee (AUP 852).

## Author contributions

DWD, WEW, and MW designed the research studies; DWD, JLY, JY, TS, SMS, SAS, EPT, YEL, and HL conducted the experiments; DWD analyzed the data; and DWD, WEW, and MW wrote and reviewed the manuscript.

## Figures and Tables

**Table 1:**
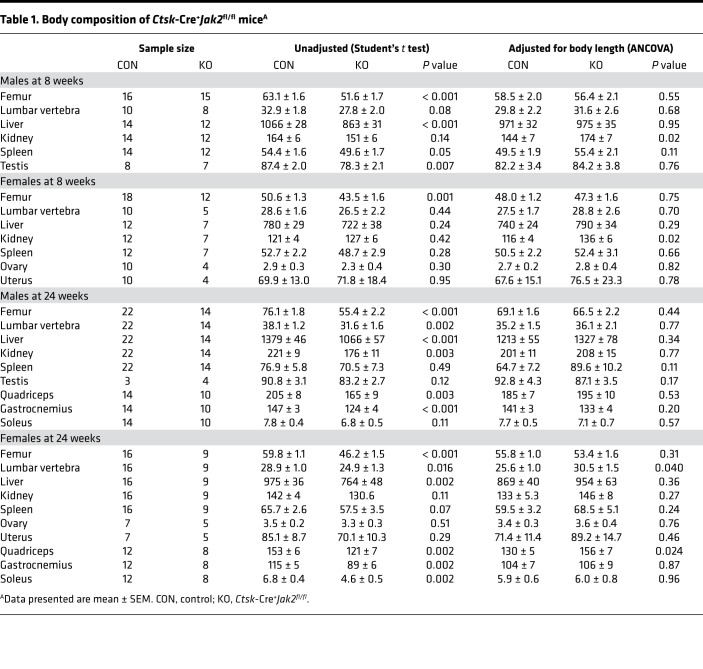
Body composition of *Ctsk*-Cre^+^*Jak2*^fl/fl^ mice^A^

**Table 2:**
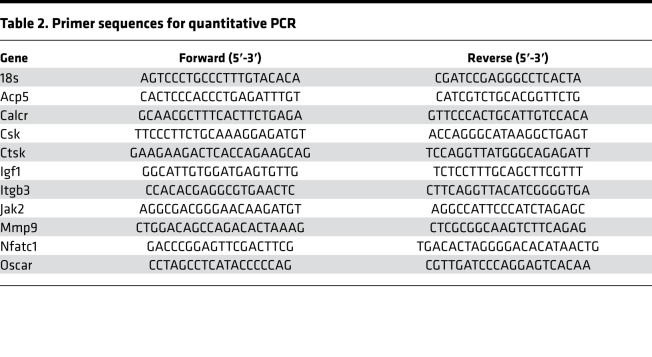
Primer sequences for quantitative PCR

## Version 1 03/08/2021

Electronic publication
